# Assessing webcam-based eye-tracking during comic reading in the classroom: a feasibility study

**DOI:** 10.31744/einstein_journal/2025AO0911

**Published:** 2025-03-28

**Authors:** Jade Antunes Nascimento, Paulo Rodrigo Bazán, Raymundo Machado de Azevedo, Edilene Santos Silva, Daniela Arruda Soares, Joana Bisol Balardin, Edson Amaro

**Affiliations:** 1 Brain Istitute Hospital Israelita Albert Einstein São Paulo SP Brazil Brain Istitute, Hospital Israelita Albert Einstein, São Paulo, SP, Brazil.; 2 Multidisciplinary Health Institute Universidade Federal da Bahia Vitória da Conquista BA Brazil Multidisciplinary Health Institute, Universidade Federal da Bahia, Vitória da Conquista, BA, Brazil.

**Keywords:** Eye-tracking technology, Usability evaluation, Comics, Method comparison, Reading patterns

## Abstract

**Objective:**

To evaluate the feasibility and accuracy of webcam-based eye tracking for monitoring comic books reading behavior in a real-world classroom setting.

**Methods:**

We tested the feasibility of using the webcam-based eye tracking in a children’s classroom experiment (n=22), observing reading patterns as students engaged with a comic strip. Qualitative quality control was implemented to exclude low-quality data. Fixation dwell time was measured to determine whether specific areas of the image received more attention. Additionally, a validation test was conducted (n=3) to assess the accuracy and precision of webcam-based eye tracking compared with an infrared-based eye-tracking system. The accuracy and precision were evaluated, with lower values indicating better performance.

**Results:**

During the comic reading task, webcam-based eye tracking effectively captured fixation dwell time, showing that students read the left side significantly faster (2.916 words/s, SD=0.705) compared to the right side (1.962 words/s, SD=0.450, p≤0.001). The validation test showed that webcam-based eye tracking had an average accuracy of 11.581% and a precision of 3.058%, whereas the infrared-based system recorded an accuracy of 11.290% and a precision of 1.264%. Although webcam-based eye tracking demonstrated a slightly lower precision (bias=-1.794, 95%CI=-3.055 to -0.532), no significant difference was observed in accuracy between the two systems.

**Conclusion:**

Despite significant data loss due to low-quality recordings, webcam-based eye tracking proved to be a feasible tool for monitoring comics reading behavior in real-world classroom settings, providing accuracy comparable to that of IR-based systems and valuable insights into students’ reading patterns.

## In Brief

Webcam-based eye tracking offers a practical approach for monitoring reading behavior in classroom environments. Nascimento et al. demonstrated the feasibility of using the Webcam-based eye tracking to observe comic reading patterns among children and showed that it effectively captures fixation time and reading dynamics. Despite the data quality challenges, Webcam-based eye tracking provided an accuracy comparable to that of infrared-based systems.

## Highlights

■ Webcam-based eye tracking demonstrated feasibility for monitoring reading behavior in classrooms.

■ Webcam-based eye tracking captured fixation times, showing differing reading speeds across comic sections.

■ Webcam-based eye tracking showed accuracy comparable to infrared-based systems, despite lower precision.

■ Limitations resulting in data loss underscore the need for methodological improvements.

## Graphical abstract

**Figure:**
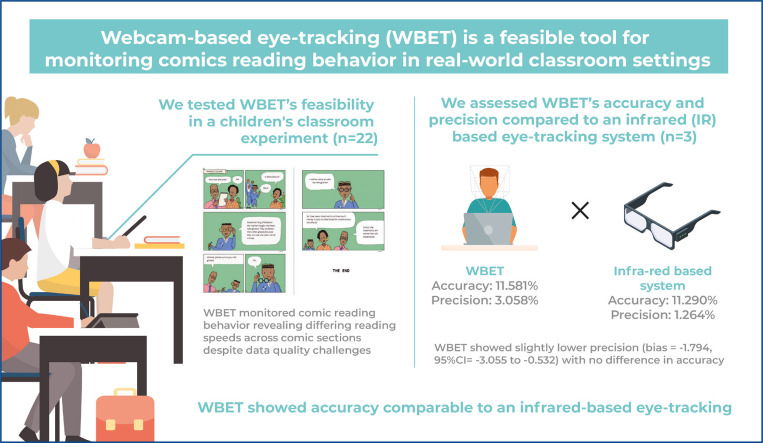

## INTRODUCTION

The recent global health crisis caused by the coronavirus disease (COVID-19) outbreak has resulted in researchers worldwide adapting their data collection methodologies for scientific investigations.^1^ Considerations have been made to ensure the continued productivity of research involving human participants amidst the reality of social distancing measures, including in schools. A viable approach to preventing in-person interactions and following social distancing guidelines involves leveraging widely accessible electronic devices for data acquisition. This entails transitioning to online interviews and questionnaires, employing telemedicine techniques, and gathering data remotely.^2-4^The utilization of webcams integrated into portable computers has fostered the interest of researchers engaged in remote data collection, particularly those employing eye tracking methodologies.^5^

Eye-tracking enables the detection and recording of ocular behavior, which has captured the interest of psychologists and neuroscientists since the proposition of eye movement as a dynamic indicator of attention direction in the 1980s.^6^Gaze, which is directly linked to attention, offers insights into cognitive processes, making eye tracking applicable in various fields such as psychology,^7^healthcare,^8^education,^9^marketing,^10^ and user experience.^11^Several methods for studying eye movements such as video-oculography, video-based tracking, and electro-oculography are available.^12,13^ Specialized devices used in these methods rely on infrared (IR) light reflection from the cornea, which provides complementary reference points which, when combined with the center of the pupil, enhance tracking accuracy.^14^However, eye tracking is considered a high-cost technology compared with other techniques used to measure behavior and attention.

Conventional eye-tracking systems typically require specialized hardware such as dedicated glasses or sensors. Whereas popular electronic devices, such as smartphones, tablets, laptops, and video game consoles, are equipped with built-in webcams. Thus, combining built-in webcams with algorithms and software allows the tracking of user gaze, a technique called webcam-based eye tracking (WBET), at a much lower cost. Therefore, WBET offers several advantages, including affordability and the opportunity to conduct eye-tracking studies in ecological environments such as homes or schools.^15^Additionally, the use of webcams integrated into portable computers has fostered the interest of researchers in eye tracking remote data collection.^5^

Despite its advantages, WBET has certain challenges and potential issues. Previous studies haves shown that they WBET often exhibits limited resolution and lower accuracy for motion detection than dedicated eye-tracking devices, thereby affecting the quality of the collected data.^5,16^However, WBET has some adaptable aspects that can affect the data quality, such as the camera utilized, head position, lighting conditions, and other environmental factors.^17^Moreover, webcams embedded in popular devices have low resolution, making it difficult to accurately track eye movements and even harder to obtain subtle measures such as saccades and pupil dilatation.^16,18^

Therefore, further testing and validation of WBET is necessary to address these challenges^19^and assess the reliability and validity of the measurements obtained using this method. Consequently, it is essential to compare the data obtained from webcam-based and specialized technologies to identify the limitations of the webcam method.^5,20^Furthermore, performing more studies using WBET can help understand its capabilities and potential applications, which can contribute to the development of methodologies and their adoption as viable alternatives.

### Eye-tracking analysis in comic book studies

An interesting context for testing the WBET application lies in the realm of comic books. Researchers have gained valuable insights into fixation patterns and reading strategies by tracking participants’ eye movements while engaging with comics.^21,22^ This method allows for a detailed examination of the aspects of a comic page that capture readers’ attention the most^21^aiding in the analysis of comprehension and reading strategies.^23-26^ Such studies allow comics creators, publishers, and scholars to enhance visual communication^27^ in this medium as well as improve teaching resources.

Comic books are frequently selected as a means of teaching challenging concepts because of their potential to facilitate information acquisition for children.^28^ Drawings enable the reading of stories by children who have difficulties with traditional reading forms. Consequently, it can facilitate the teaching of complex concepts such as those related to critical thinking and scientific knowledge in the field of health.^28^Therefore, eye-tracking can be used as an investigation tool, as it has been used in education research.^9^

Additionally, WBET technology can be applied to analyze teaching resources in underserved communities, mainly because of its low cost. This is particularly significant for these communities as they often face financial constraints and cannot afford to spend money on materials for which they are unsure of their functionality or effectiveness. Thus, WBET allows us to evaluate the feasibility^29^ and practicality of materials in challenging educational environments. Ultimately, this contributes to enhancing educational quality and addressing student needs in underserved contexts. Furthermore, it a contributes the most to testing the feasibility and viability of WBET to replace specialized eye trackers in research studies, particularly for tracking attention in reading during remote and online experimentation.^30,31^

## OBJECTIVE

This study aimed to evaluate the feasibility of using webcam-based eye tracking in a classroom setting during comic strip reading, as a mean to evaluate teaching resources.

## METHODS

### School context

The study included 22 (10-12 years-old) children from a 6^th^ year class from a school in Vitória da Conquista, Bahia, northeastern Brazil. Informed consent was obtained from the children and their parents or guardians. This study was approved by the Research Ethics Committee of *Hospital Israelita Albert Einstein* (CAAE: 29757720.0.0000.0071; # 414,171 and SGPP 4078-20).

### Eye-tracking equipment

Two eye tracking devices were used in this study. the The GazeRecorder software was used on two notebooks for data acquisition via the webcam, in which the resolution of the cameras was 640×480 at 30fps and 1280×720 at 30fps for the validation task and comic reading task, respectively.

The IR eye-tracking data were captured using Tobii Glasses Pro II, which is a portable eye-tracking device that uses corneal reflection and dark pupil methods. The system consists of several components, each serving a specific purpose. The head unit, which is a wearable eye tracker in the form of glasses, features two sensor cameras per eye that record the eye orientation, an IR illuminator that illuminates the eyes to support the sensor cameras, and a high-definition camera that records what is in front of the user. Data is captured at a sampling rate of 100Hz and a video resolution of 1920×1080 pixels at 25fps.

### Webcam-based eye-tracking validation test

**Figure 1:**
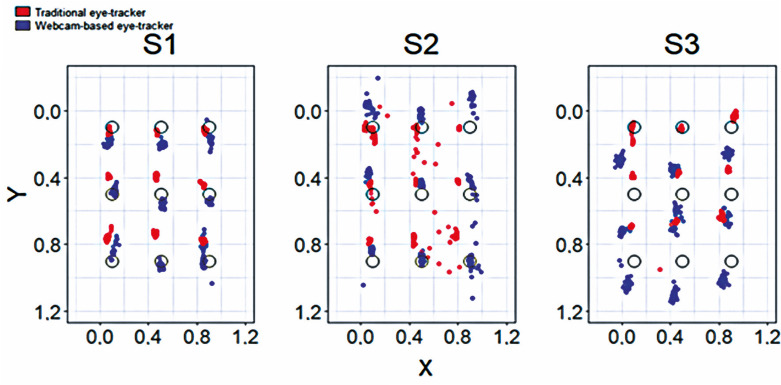
Overlapping points detected by both eye-tracking methods

S1: participant 1; S2: participant 2; S3: participant 3.

As a preliminary step, we conducted a validation test to assess the reliability of WBET used in this study. Three authors-two men and one woman-volunteered for this validation test, with ages ranging between 24-34 years. All three participants underwent the procedure without wearing glasses, including those who required vision correction lenses, to aid eye identification by the hardware. A screen with nine small black circles (reference points, Figure 1S, Supplementary Material) was shown to the participants, who received verbal instructions to look at the next reference point at intervals of approximately 3s (controlled with a stopwatch). The sequential order was followed from the top left corner to the bottom right, as that in the left to write (top to bottom) reading. Thus, the eye-tracker gaze estimate could be compared with the actual reference position. The validation task lasted approximately 30s. Each volunteer performed the task twice: once with the WBET GazeRecorder^32^ software and once with the Tobii Glasses Pro II^33^ device. Before the validation task, calibration was performed based on the eye tracker used. In the WBET, facial recognition is automatically performed by the software as the participant gazes at a red circle in the center of the screen. The calibration process involves nine red circles that appear and disappear individually at various locations on the screen. In contrast, in IR eye tracking, calibration was conducted while the participant focused on a circular figure placed at a distance of one outstretched arm.

### Comic reading task

Data were collected from a school that was accepted as a part of the Informed Health Choices (IHC) project intervention pilot in Brazil.^34^The IHC project develops teaching resources to promote citizen engagement in health decision making by teaching critical and scientific thinking.^35^These resources are currently being translated, adapted, and tested in various countries, including Brazil.^36^The eye-tracking analysis was part of a teaching resource user experience investigation.

**Figure 2:**
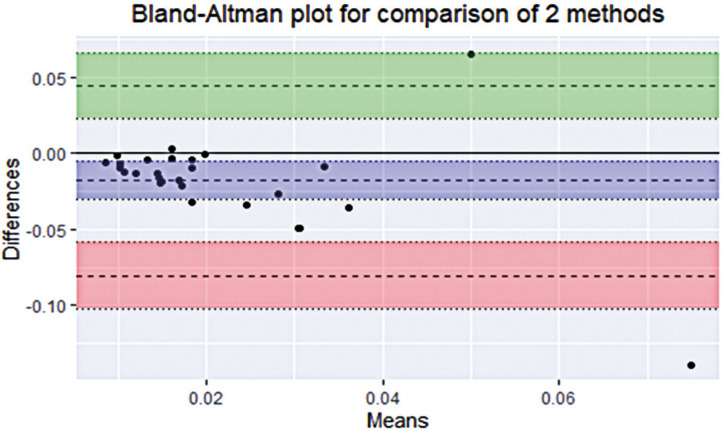
Scatter diagram of average precision by Bland-Altman test

**Figure 3:**
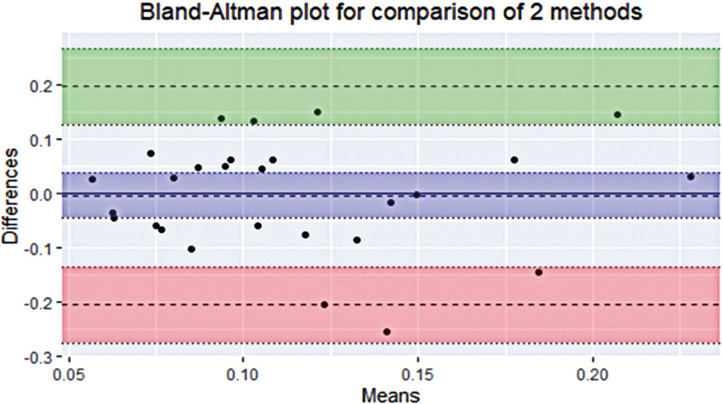
Scatter diagram of average accuracy by Bland-Altman test

Data collection was performed in the second week of classes so that the children could be exposed to the comic strip during the class in which its key concept would be taught. The students were recruited individually in another room during class to perform the task. The laptop was positioned in a bright and illuminated place in the room to optimize the eye-tracker data acquisition. The researcher performed facial recognition and the 9-point calibration with the participant. The figure was displayed on the full screen. Participants were instructed to keep their heads still and silently read the comic strip. Figure 2S, Supplementary Material shows the Brazilian Portuguese version of the comic used in the task and Figure 3S, Supplementary Material shows the original English version. The recording was performed for 1min, which is the time limit of the free version of the software.

## DATA ANALYSIS

### Webcam-based eye-tracking validation test

The acquisition software provided fixation coordinates for the time series. The validation test was performed by calculating the accuracy and precision of eye tracking. The accuracy was calculated as the average Euclidean distance (as a percentage of the test image size) between the fixations detected by the eye tracker and the reference points. Precision was calculated as the standard deviation of the Euclidean distance between the detected fixation and reference points. Values closer to zero indicate better accuracy and precision. These metrics were calculated per reference point, and the resulting nine values were averaged to obtain the participants’ accuracy and precision values. The participants’ average accuracy indicates that, on average, the measurements of the participants were x% away from the reference points. The average precision reflects the dispersion between measurements.

In IR-based eye-tracking data, a planar homography transformation is required to transform the data from the video coordinates of the glasses to test the image (computer screen) coordinates. This allows the calculation of accuracy and precision to be carried out as a percentage of screen coordinates, as in WBET. Planar homography describes the relationship between two views of the same planar surface by converting points from one view to another assuming that the observed scene lies on a flat, two-dimensional plane.^37^The homography was estimated by matching four points (the corners of Figure 1) in the eye tracking videos were recorded over time. The locations of the four points were manually determined throughout the video for all participants.

The Bland-Altman test^38^ was performed to assess the concordance between the two acquisition methods as it calculates the mean difference between two measurement methods (the bias) and the 95% limits of agreement as the mean difference ±1.96 standard deviations. The 95% limit of agreement was expected to encompass 95% of the differences between the two measurement methods. The values used were the accuracy and precision means for all nine reference points of the three participants, resulting in 27 values.

### Comic reading task

The eye-tracking assessment was described by the central and dispersion tendencies of the reading speed measures. The Wilcoxon’s^39^ test was used to compare the subject’s data. Kernel Density Estimation^40^ was used to create heat maps. Density was calculated based on the number of points in a location, with larger numbers of clustered points resulting in yellow regions on the map and small numbers resulting in blue regions.

The WBET software automatically generated heatmap videos, where the heated colors illustrated the locations of the participants’ gazes over time. During the quality control process, the heatmap videos were qualitatively analyzed, leading to the exclusion of data from ten participants owing to poor quality. In this context, poor quality refers to data that do not accurately reflect participants’ gaze behavior. Specifically, some recordings lacked any observable density on the heat maps, while in others, gaze density appeared momentarily but vanished suddenly. In addition, the raw eye-tracking data indicated that some participants directed their gaze toward areas far from the laptop screen. However, concurrent video recordings captured by the webcam during the task showed that these participants were actually looking at the screen for the entire duration. This discrepancy between eye tracking data and video evidence underscores the unreliability of the data from these participants. Consequently, these data points were excluded to preserve the integrity of our analysis, preventing them from being included in the data analysis process.

## RESULTS

### Webcam-based eye-tracking validation test

**Figure 4:**
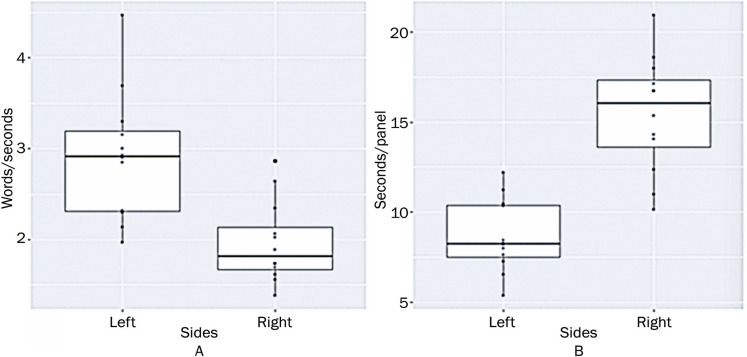
Comic strip reading speed (words/sec) and dwell time per panel (sec/panel)

The WBET validation tests were performed by three authors. Figure 1 shows the overlapping distribution of screen fixations detected by both methods on the calibration image for the three participants. The red dots represent the fixations detected by the IR-based eye tracker and the blue dots represent the fixations detected by WBET. Figures 4S and 5S, Supplementary Material show the distribution of fixations on the screen for the three participants with WBET-and IR eye trackers, respectively. The x- and y-axes represent the surfaces viewed by the participants as percentages (from 0 - 1). The image displayed during data collection was inserted inside the area formed by coordinates (0.0), (0.1), (1.1), and (1.0). The highlighted dots represent the nine reference points displayed to the participants during the test. Colored point clouds depict the fixations assigned to each reference point of the same color. The point clouds represent the locations where the WBET detected that the participants fixated on their gazes while staring at the reference points.

Based on the figures, the IR eye-tracker performed more accurately at the upper points along the y-axis but gradually worsened at the lower points. The eye-tracking data suggested that Participant 1 (S1) exhibited greater accuracy in both data collection tasks. Participant 2 (S2) exhibited the worst accuracy and precision for both data collection methods.

The average accuracy and precision were 11.581% and 3.058%, respectively, for WBET. The accuracy and precision of the IR-based eye tracker were 11.290% and 1.264%, respectively. In general, the participants did not show significant differences between the methods, with the same participant having the best accuracy in both methods, while another participant was having the worst accuracy in both the methods. Additionally, both methods presented better precision than accuracy. Table 1 presents the average precision and accuracy across the reference points for each participant for both data collection methods.

**Table 1:** Measures of accuracy and precision from both eye-trackers

Participant	Webcam-based eye-tracker		Infra-red based eye-tracker	
	Average accuracy (%)	Average precision (%)	Average accuracy (%)	Average precision (%)
1	7.053	3.580	9.564	0.818
2	8.812	2.624	10.510	2.088
3	18.877	2.969	13.797	0.885
Mean	11.581	3.058	11.290	1.264

The results of the Bland-Altman test for the 27 values of average precision and accuracy are illustrated in figures 2 and 3, respectively, and table 2. A slight negative bias was observed in the precision, indicating that the precision measures were better in the IR-based eye tracker. This is indicated by the fact that zero is outside the 95% confidence interval in figure 2. However, the bias was close to zero in the comparison of average accuracy, implying that the two methods produced similar accuracy results. The value exceeded the minimum acceptable difference between the methods in only one of the observations of the precision comparison.

The middle horizontal dotted line is the bias and refers to the mean of the differences of the two measurements. The blue interval refers to the 95% confidence interval of the mean difference. The upper horizontal dotted line is the Upper LoA and represents the average difference + 1.96 times the standard deviation of the difference. The lower horizontal dotted line is the Lower LoA and represents the average difference - 1.96 times the standard deviation of the difference. The intervals in green and red represent the 95% confidence interval of LoA, demonstrating the error or precision of the upper and lower LoA respectively

The middle horizontal dotted line is the bias and refers to the mean of the differences of the two measurements. The blue interval refers to the 95% confidence interval of the mean difference. The upper horizontal dotted line is the Upper Limit of Agreement (LoA) and represents the average difference + 1.96 times the standard deviation of the difference. The lower horizontal dotted line is the Lower LoA and represents the average difference - 1.96 times the standard deviation of the difference. The intervals in green and red represent the 95% confidence interval of LoA, demonstrating the error or precision of the upper and lower LoA respectively

**Table 2:** Bland-Altman test results for the two methods comparison

	Accuracy means (%)	Precision means (%)
Bias (95%CI*)	-0.290 (-4.346 to 3.765)	-1.794 (-3.055 to -0.532)
Standard error of bias	1.973	0.614
Upper limit of agreement (95%CI)	19.805 (12.786 to 26.824)	4.456 (2.273 to 6.639)
Lower limit of agreement (95%CI)	-20.386 (-27.404 to -13.367)	-8.044 (-10.227 to -5.861)

*The 95% confidence interval (95%CI) establishes the range in which, approximately 95% of the time, differences in data from one technique to another will be found.

### Eye tracking task

Eye-tracking data were obtained from 12 participants while they read a comic strip from the Health Decisions Book. The values of gaze duration on the left and right sides of the image and gaze duration per panel in seconds were obtained using the gaze location time series, along with the reading speed of the participants (words per second), as shown in table 3. The mean, standard deviation, and median values were also calculated.

**Table 3:** Gaze dwell time and reading speed on the comic strip

Participant	Left - time (s)	Right - time (s)	Total - time (s)	Left - time (s/panel)	Right - time (s/panel)	Total - time (s/panel)	Left - speed (word/sec)	Right - speed (word/sec)	Total - speed (word/sec)
1	24.016	30.728	54.744	8.005	15.364	10.949	2.998	1.887	2.375
2	31.343	28.087	59.430	10.448	14.043	11.886	2.297	2.065	2.187
3	22.815	37.185	60.000	7.605	18.592	12.000	3.156	1.560	2.167
4	24.583	20.250	44.833	8.194	10.125	8.967	2.929	2.864	2.900
5	24.839	34.274	59.113	8.280	17.137	11.823	2.899	1.692	2.199
6	25.297	33.405	58.703	8.432	16.702	11.741	2.846	1.736	2.214
7	21.833	35.984	57.817	7.278	17.992	11.563	3.298	1.612	2.248
8	33.704	24.749	58.453	11.235	12.374	11.691	2.136	2.343	2.224
9	36.639	21.967	58.606	12.213	10.983	11.721	1.965	2.640	2.218
10	31.029	28.603	59.632	10.343	14.301	11.926	2.320	2.028	2.180
11	16.120	41.855	57.976	5.373	20.927	11.595	4.466	1.386	2.242
12	19.526	33.441	52.967	6.509	16.720	10.593	3.687	1.734	2.454
Mean	25.979	30.877	56.856	8.659	15.439	11.371	2.916	1.962	2.301
Standard deviation	6.0393	6.423	4.3132	2.013	3.211	0.863	0.705	0.450	0.207
Median	24.711	32.066	58.530	8.237	16.033	11.706	2.914	1.812	2.221

The average reading speed on the left side of the image was 2.9 words per second (175.0 words per minute) and on the right side was 2.0 words per second (117.7 words per minute). The Wilcoxon test showed a statistical difference between the two medians (p-value=4.96 x 10^4). The average gaze duration per panel on the left side was shorter (8.659 s/panel) than that on the right side (15.439 s/panel). A significant difference was also observed between the gaze duration per panels as showed by the Wilcoxon test (p-value=2.219 × 10^-5) (Figure 4).

A) Distribution of the participant’s reading speed on the left and right sides of the image; B) Distribution of the participant’s gaze duration per panel on the left and right sides of the image.

Heatmaps (Figures 6S to 8S, Supplementary Material) were obtained for the 12 participants (S1-S12). The level color ranged from blue to yellow, in which the shades of yellow represent the places where the participant’s gaze remained for the longest time, and the shades of blue represent the places where the participant’s gaze remained for a shorter time.

## DISCUSSION

Based on the results of this study, webcam-based eye tracking technology appears to be a highly promising method in classroom settings, as it was proven to be useful in this assessment through validation tests and actual tasks.

### Webcam-based eye-tracking validation test

The Bland-Altman test employed in this study demonstrated that both the IR-based and WBET methods exhibited a high level of concordance. Although the IR-based eye tracker exhibited greater precision according to the proximity of the accuracy measures, increasing the sample size and the lower precision of the WBET had a reduced impact. Therefore, we conclude that WBET can effectively serve as an eye-tracking data collection method under similar conditions.

The precision and accuracy measures of the WBET method can be considered high, as indicated by the 15% screen size accuracy and 4% precision found in similar fixation tasks.^5^In contrast, precision and accuracy measurements for IR-based eye trackers are not as accurate because of the need to transform the fixation coordinates to the 2D plane through planar homography, adding errors to these measurements. Another results that can be attributed to the errors caused by the planar homography transformation is that the IR eye tracker performed more accurately at the upper points along the y-axis and gradually worsened at the lower points. This variation could also be attributed to other factors, such as the position of the head unit (glass structure), which can be more precise and accurate in the centerline of vision. However, further analyses are required to determine the exact cause of this discrepancy. Moreover, participant 1 also exhibited greater accuracy in both data collections, whereas Participant 3 (S3) exhibited the worst accuracy in both collections. This could be related to individual aspects, such as the participant’s eye color or head movements.

The validation test results corroborate the previous findings, which indicate that WBET technology can achieve the same performance as IR-based eye trackers.^20^ However, the primary limitation of WBET eye tracking is its lack of sensitivity in detecting movements compared to specialized equipment, making it more suitable for situations where high precision is not required, such as in large areas of interest.^20,41^

Nonetheless, utilizing eye tracking via webcams offers numerous benefits, including expanded data collection capabilities and the ability to conduct research remotely because of the widespread availability of webcams on digital devices.^42^ However, limitations include camera quality, frame rate, and the absence of infrared light sources, which can affect accuracy owing to interference from external light sources.^12,43,44^ Thus, many researchers have adopted the WBET technology in their studies, often comparing it to IR devices to ensure reliability.^5,15,45^

### Comic book reading task

Among the 22 eye-tracking recordings, 12 yielded high-quality data, highlighting the feasibility of using WBET to track student attention while reading comic stories in a classroom setting. Data analysis revealed that it is possible to determine the location of an individual’s gaze on each side of the image and find significant differences in the speed of comic reading using this method, as explored in the following paragraphs. However, we believe that the loss of quality in the other 10 recordings may be due to potential factors such as participants wearing glasses in a bright room, which caused reflections on the lenses and occasionally led the software to lose track of their gazes. Additionally, students’ head movements or the researcher positioning herself in front of the notebook to start the recording were considered possible reasons for interference in gaze recognition. These results are consistent with a previous eye-tracking feasibility study,^46^wherein data loss was highlighted as one of the key lessons learned, attributed to factors such as inconsistent lighting, temporary hardware malfunctions, minor eye movements, and wearing of masks and eyeglasses. Moreover, our results are consistent with those of other studies that have tested WBET models in real-world settings, also finding evidence of their feasibility, although highlighting significant room for improvement.^47^ In this study, the students exhibited a higher average reading speed on the left side of the comic book, indicating a faster reading of the story’s beginning. This suggests that the later panels may demand a greater cognitive load for comprehension, as the time spent on each panel is correlated with the cognitive effort required to process the information.^9,14^ The reason for this may be that the concept’s explanation occurs in the last panel, which may have led to a moment of greater reflection by the students in that part of the story.

Notably, the overall average reading speed was lower than the global averages, possibly influenced by pandemic-related school closures shutting down students’ learning environments.^48^ In addition, although Latin languages seems to have a higher average reading speed than other languages,^48^a study in including fifth-grade Brazilian students also demonstrated low reading speeds.^49^

The eye-tracking heat maps did not reveal a consistent reading pattern or fixation location among participants, likely because of the less constrained nature of image and comic processing compared with text, in which readers are expected to read word-by-word and line-by-line. Comics afford readers more freedom to explore content^24^ and participants’ interests in specific images or panels may vary individually. However, it was possible to identify the horizontal gaze direction, *i.e*., the time at which the participants read the left or right part of the image. The use of WBET, which provides lower image quality than specialized eye tracking^44^ further complicates the analysis of which panel each participant remained on for the longest time.

Despite its positive outcomes, this study has certain limitations, to including the data collection process. Since the study was conducted in a school room, people entered and left the room at some time, which can be considered a distracting factor. However, the advantage of conducting data collection at school lies in the ecological context, as students are simultaneously engaged in nearby classes.

## CONCLUSION

This study highlights the potential of the WBET technology as an accessible tool for classroom research. Despite its slightly lower precision compared to infrared-based systems, WBET proved effective in tracking gaze behavior during comic reading tasks, revealing meaningful attention patterns. Limitations, such as data loss, environmental distractions, and lower image quality, underscore the need for methodological improvements. However, the ecological validity of conducting research in a natural classroom setting demonstrated of WBET as a cost-effective alternative for educational studies. Future studies should refine its accuracy and explore broader applications in learning environments.
