# Supplementary material for: Assessing webcam-based eye-tracking during comic reading in the classroom: a feasibility study
Source: Einstein (Sao Paulo). 2025 Mar 28;23:eAO0911. doi: 10.31744/einstein_journal/2025AO0911 (PMC12014154; doi:10.31744/einstein_journal/2025AO0911)
Supplement: Supplementary file 1 [file 2317-6385-eins-23-eAO0911-suppl01.pdf]

SUPPLEMENTARY MATERIAL

Assessing webcam-based eye-tracking during comic reading in the classroom: a feasibility study

Jade Antunes Nascimento, Paulo Rodrigo Bazán, Raymundo Machado de Azevedo Neto, Edilene Santos Silva, Daniela Arruda Soares, Joana Bisol Balardin, Edson Amaro Júnior

DOI: 10.31744/einstein_journal/2025AO0911

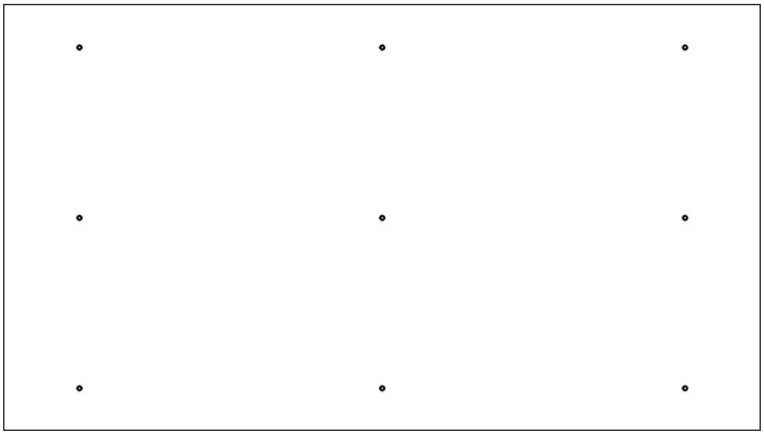

Figure 1S. Reference points image used for WBET validation test

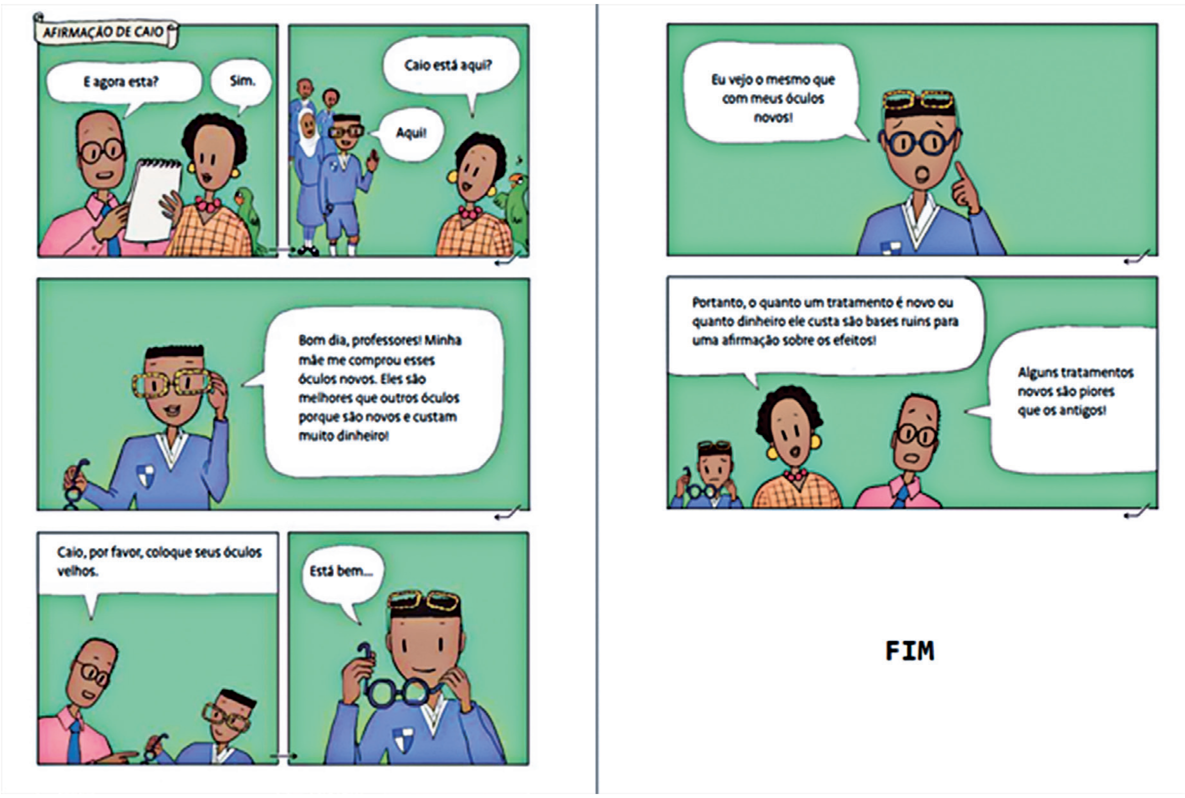

Figure 2S. Comic strip from the Brazilian Portuguese version of the Health Choices Book (Children's Book)

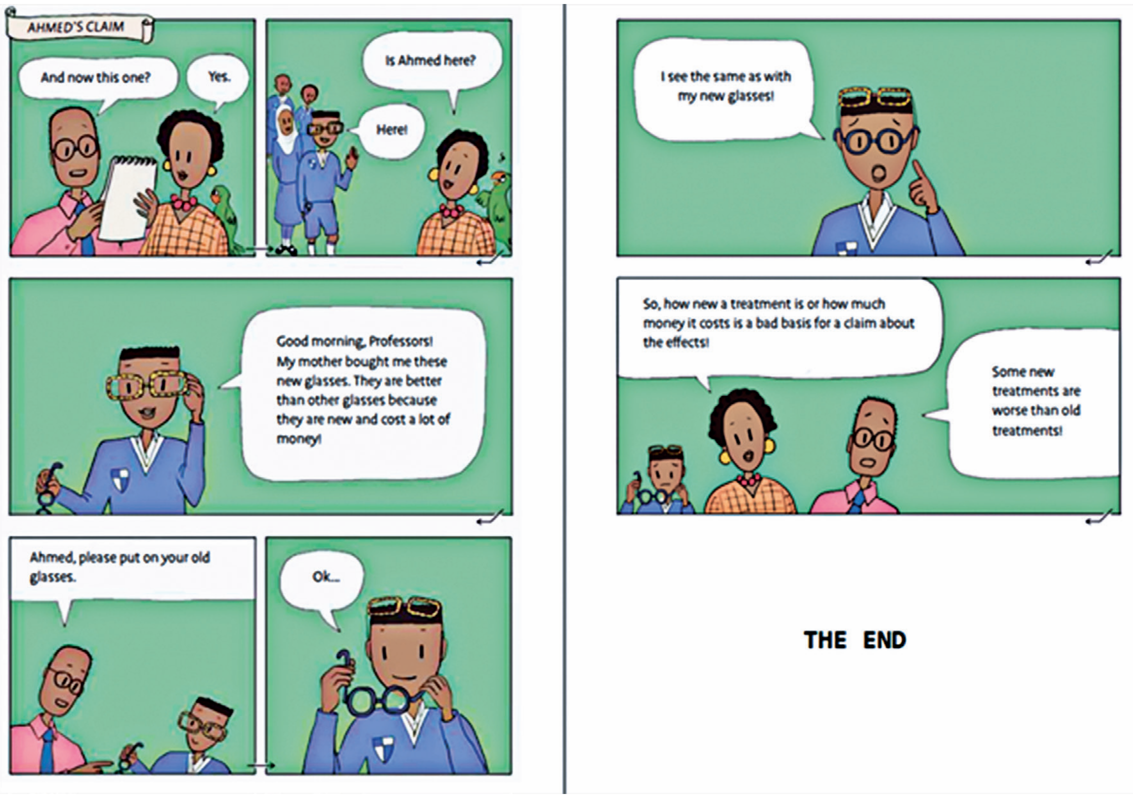

Figure 3S. Comic strip from the English version of the Health Choices Book (Children's Book)

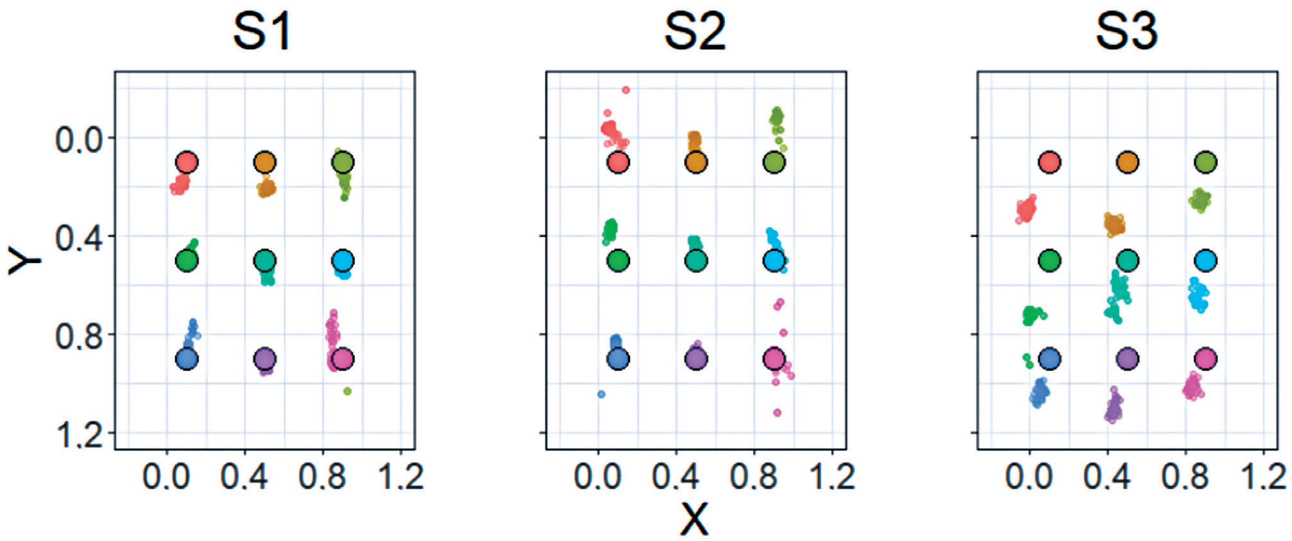

Figure 4S. Distribution of fixations detected by the WBET during the calibration test. Participants: S1 (Participant 1), S2 (Participant 2), and S3 (Participant 3)

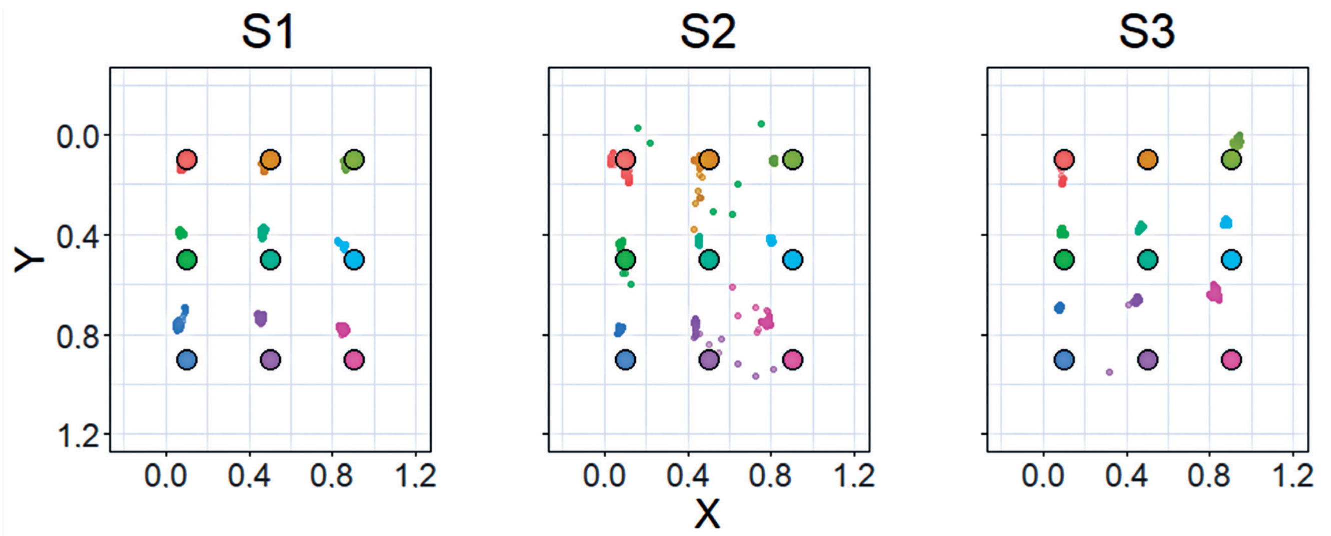

Figure 5S. Distribution of fixations detected by the IR-based eye tracker during the calibration test. Participants: S1 (Participant 1), S2 (Participant 2), and S3 (Participant 3)

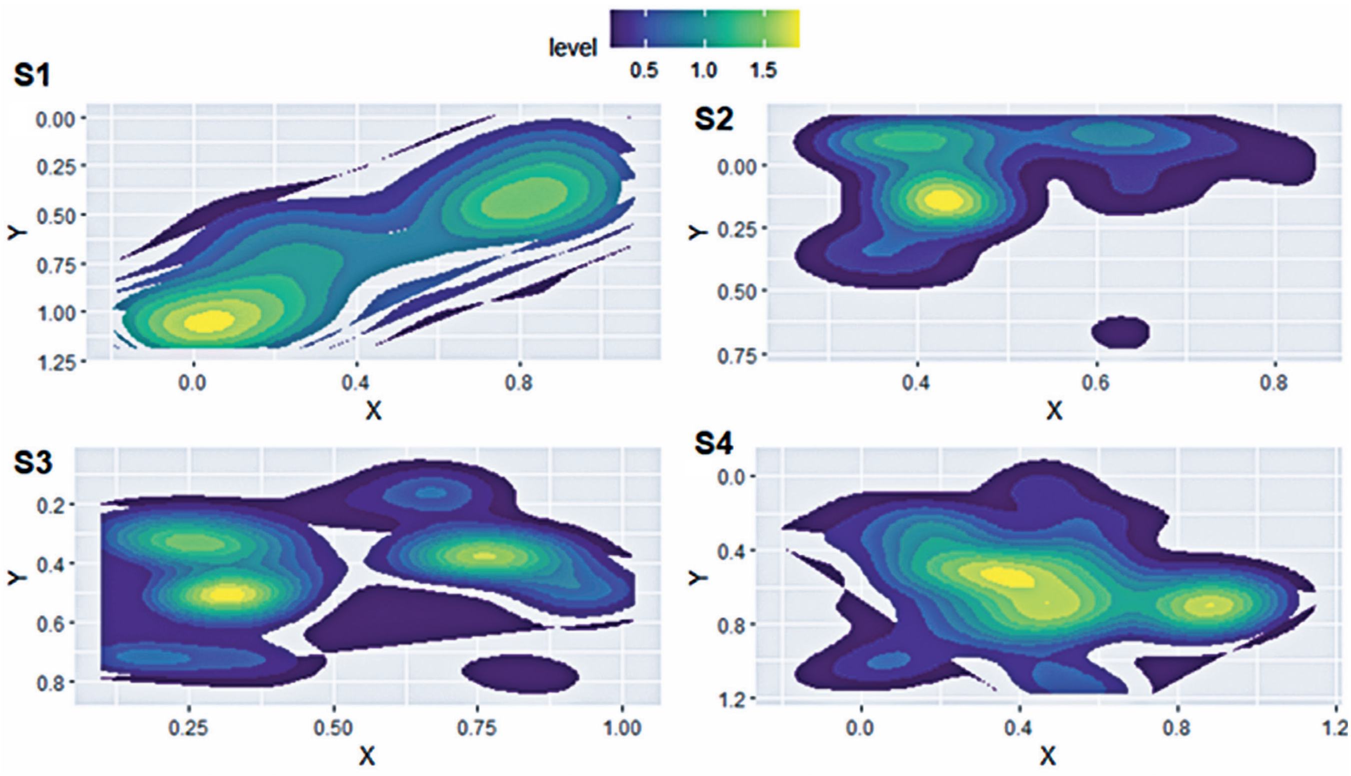

Figure 6S. Gaze permanence heatmap for participants S1-S4 (S1: Participant 1, S2: Participant 2, S3: Participant 3, S4: Participant 4). Higher levels indicate regions with a higher density of fixations. The X and Y axes represent fixation coordinates, proportionate to the image size (0,0=top left; 1,1=bottom right)

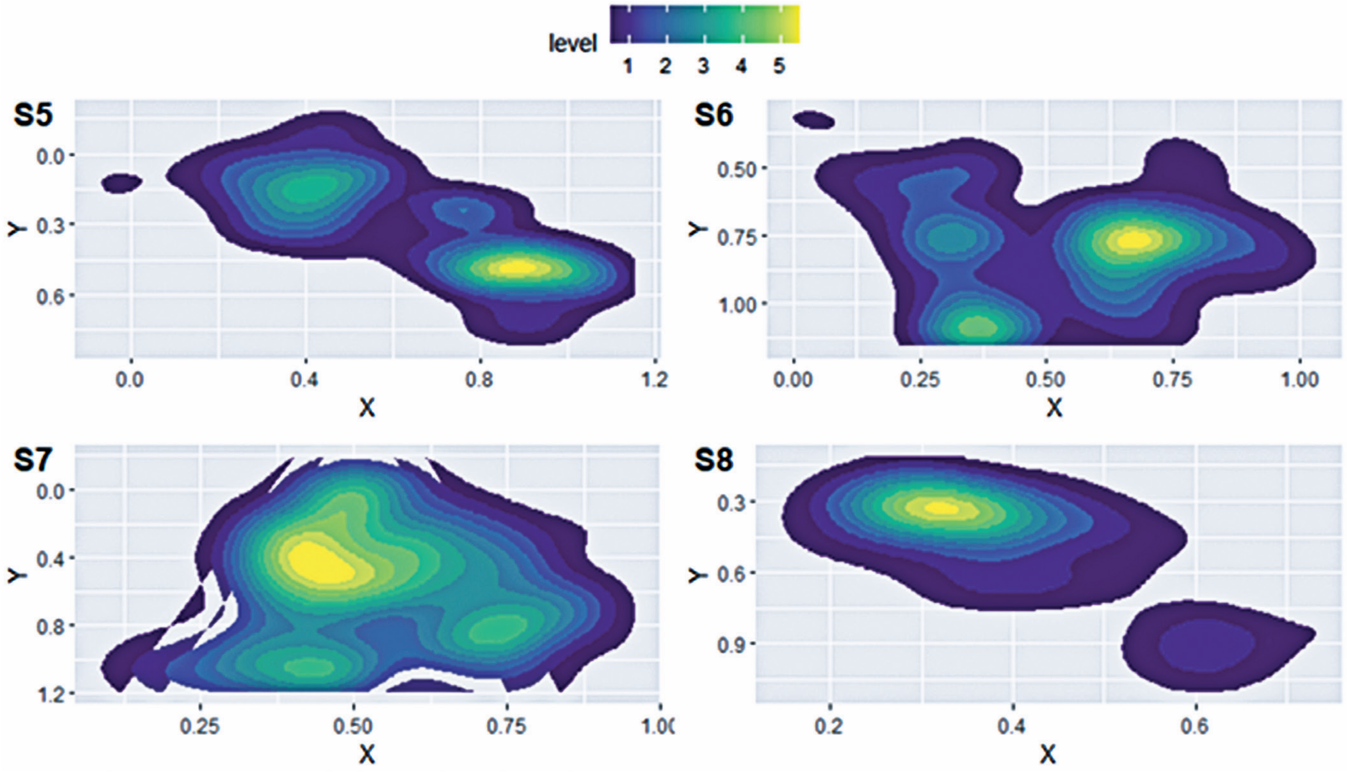

Figure 7S. Gaze permanence heatmap for participants S5-S8 (S5: Participant 5, S6: Participant 6, S7: Participant 7, S8: Participant 8). Higher levels indicate regions with a higher density of fixations. The X and Y axes represent fixation coordinates, proportionate to the image size (0.0=top left; 1.1=bottom right)

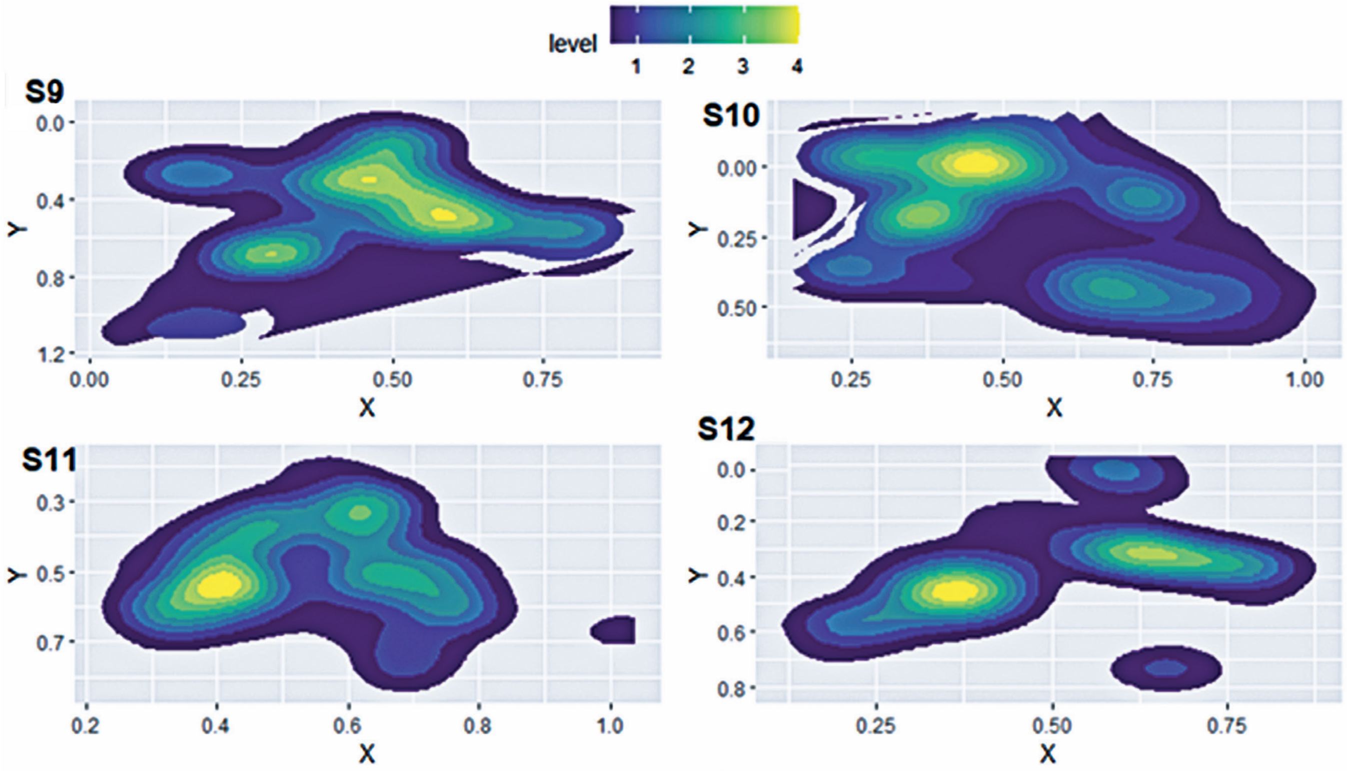

Figure 8S. Gaze permanence heatmap for participants S9-S12 (S9: Participant 9, S10: Participant 10, S11: Participant 11, S12: Participant 12). Higher levels indicate regions with a higher density of fixations. The X and Y axes represent fixation coordinates, proportionate to the image size (0.0=top left; 1.1=bottom right)
